# Rapid protection against viral infections by chemokine-accelerated post-exposure vaccination

**DOI:** 10.3389/fimmu.2024.1338499

**Published:** 2024-01-29

**Authors:** Annkristin Heine, Niels A. W. Lemmermann, Chrystel Flores, Janine Becker-Gotot, Natalio Garbi, Peter Brossart, Christian Kurts

**Affiliations:** ^1^ Institute of Experimental Immunology, University of Bonn, Bonn, Germany; ^2^ Medical Clinic III, University of Bonn, Bonn, Germany; ^3^ Institute for Virology and Research Center for Immunotherapy (FZI) at the University Medical Center of the Johannes Gutenberg University Mainz, Mainz, Germany; ^4^ Institute for Virology, University of Bonn, Bonn, Germany; ^5^ Doherty Institute for Infection and Immunity, University of Melbourne, Melbourne, VIC, Australia

**Keywords:** chemokines, CTL induction, post-exposure vaccination, murine cytomegalovirus, viral infection, NKT cells, TLR ligand, adenovirus

## Abstract

**Introduction:**

Prophylactic vaccines generate strong and durable immunity to avoid future infections, whereas post-exposure vaccinations are intended to establish rapid protection against already ongoing infections. Antiviral cytotoxic CD8^+^ T cells (CTL) are activated by dendritic cells (DCs), which themselves must be activated by adjuvants to express costimulatory molecules and so-called signal 0-chemokines that attract naive CTL to the DCs.

**Hypothesis:**

Here we asked whether a vaccination protocol that combines two adjuvants, a toll-like receptor ligand (TLR) and a natural killer T cell activator, to induce two signal 0 chemokines, synergistically accelerates CTL activation.

**Methods:**

We used a well-characterized vaccination model based on the model antigen ovalbumin, the TLR9 ligand CpG and the NKT cell ligand *α*-galactosylceramide to induce signal 0-chemokines. Exploiting this vaccination model, we studied detailed T cell kinetics and T cell profiling in different *in vivo* mouse models of viral infection.

**Results:**

We found that CTL induced by both adjuvants obtained a head-start that allowed them to functionally differentiate further and generate higher numbers of protective CTL 1-2 days earlier. Such signal 0-optimized post-exposure vaccination hastened clearance of experimental adenovirus and cytomegalovirus infections.

**Conclusion:**

Our findings show that signal 0 chemokine-inducing adjuvant combinations gain time in the race against rapidly replicating microbes, which may be especially useful in post-exposure vaccination settings during viral epi/pandemics.

## Graphical Abstract

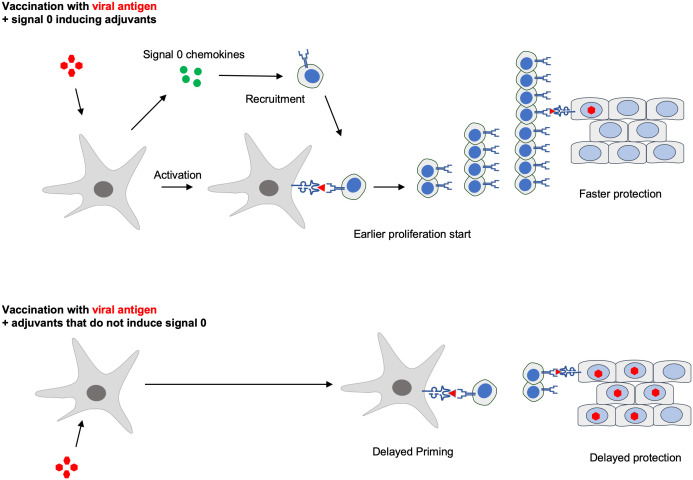

## Introduction

Prophylactic vaccines are designed to establish strong and durable immunity against future infections, usually by the induction of neutralizing antibodies. The speed by which protective immunity is established represents a distinct vaccine quality that is of minor importance for prophylactic vaccinations. However, it is critical for post-exposure vaccines that are intended to establish rapid protection after exposure to infectious agents. This is especially important for prophylactic antiviral treatment of immunocompromised patients and in areas where viral infections are epidemic, such as hemorrhagic fever in central Africa (1–3).

The most important immune cells in early control of viral infection are cytotoxic CD8^+^ T cells (CTL) which can recognize and kill virus-infected cells. They are induced within days after infection, and thus much earlier than antibodies, which require weeks for optimal effectivity. Hence, CTL can contain viral infections before neutralizing antibodies have been generated. Naïve CTL are activated by dendritic cells (DCs) that present viral antigenic peptides on MHC I molecules (4). Antigens from vaccines or from viruses that do not infect DCs are presented by a distinct DC subset, and the resultant CTL activation has been termed cross-priming (5). Cross-priming is important for CTL responses against viruses that evade the MHC class I presentation pathway, such as members of the herpes virus family (6). An important example is human cytomegalovirus infection, which can cause life-threatening disease in immunocompromised patients (7–9).

The classic Bretscher/Cohn model of naïve T-cell activation proposed that in addition to antigen recognition (signal 1), a costimulatory signal is required to induce immunity (10). DCs express this signal 2 after sensing molecular patterns indicating microbial infection or danger (11). Effective vaccines contain adjuvants that induce signal 2, for example, ligands of pattern recognition receptors like Toll-like receptors (TLR) (12, 13). Optimal CTL cross-priming also requires signals from helper immune cells, classically CD4^+^ T helper (Th) cells, which can “license” the DCs (14). Licensing requires physical encounter of cross-presenting DCs, antigen-specific CTL, and antigen-specific Th cells. To facilitate such improbable tripartite encounters, Th cells and TLR-ligands stimulate DCs to produce chemokines binding the CCR5 receptor, which attract CTLs toward the licensed DCs (15, 16), thereby avoiding time-consuming interaction with unlicensed DCs that lack relevant antigen presentation. Natural killer T (NKT) cells can cause an alternative type of DC licensing (17). NKT cells recognize glycolipid antigens presented by DCs on the MHC-I like molecule CD1d (18) and release the chemokine CCL17, that recruit CTL through CCR4 (17). As CCR4- and CCR5-chemokines act before signals 1 and 2, they have been termed “signal 0” in T cell activation (19).

Several studies examined how adjuvants affect the strength and duration of vaccine-induced immune responses (20–24). The potential of signal 0 chemokines for vaccination improvement is currently unclear. Late after vaccination, i.e. in the CTL effector phase, topically applied chemokines directed previously primed CTL into sites infected by genital herpes simplex virus infection and established local defense, termed “prime-and-pull” vaccination (25). Also in the CTL priming phase, a positive effect of chemoattractants has been predicted by mathematical models, which proposed that chemotactic migration of T cells toward DCs may promote the efficient detection of rare antigens (26). Here we verified this notion by showing that adjuvants that induce CCR4- and CCR5-chemokines can synergistically accelerate CTL activation and establish faster antiviral protection.

## Materials and methods

### Reagents, mice, animal keeping

C57BL/6(N) mice were bought from Janvier or Jackson Laboratories. Knockout mice were bred at the central animal facility of the University Hospital of Bonn. All mice had been backcrossed to C57BL/6(N) at least ten times, were bred under specific pathogen-free conditions, and were used at 8–12 weeks of age. For all experiments, mice were sex-, age-, and/or weight-adjusted. Experiments were performed according to the NIH Guide for the Care and Use of Laboratory Animals. *In vivo* experiments were approved by the Local Animal Care Commission of North Rhine Westphalia (permit number 84-02.04.2016.A102 and 84-02.04.2014.A140) or by the ethics committee of the Landesuntersuchungsamt Rheinland-Pfalz (permit number 177-07/G11-1-004). All reagents were from Sigma-Aldrich unless otherwise specified. Soluble ovalbumin (OVA; 10 μg per gram body weight) was injected intravenously or subcutaneously in a total volume of 100 μl, accompanied, when appropriate, by 0.2 μg of *α*-galactosylceramide (αGC) (1 nmol, Enzo Life Science) or/and 20 μg of CpG ODN 1668 (TBI Mol Berlin). NK and CD8 T-cell depletion was performed as described previously (27) with anti-asialo GM1 (20 μl in 200 μl of PBS i.v., WAKO Chemicals) and with the monoclonal antibody YTS169.4 (BioXCell) (28) directed against CD8, respectively. Isotype controls were from BioXCell.

### Knockout models

As CCL17-deficient mice, homozygous CCL17- eGFP knock-in mice were applied in which both Ccl17 genes were replaced with a gene encoding enhanced green fluorescent protein (eGFP), leaving the Ccl17 promoter in place (17, 29). These were a kind gift of Prof. Irmgard Förster, LIMES Bonn, Germany. CCR5KO and CCR4KO mice were obtained from JAX Mices & Services. CCR5KO-OT-Is were obtained by crossing CCR5KO mice to C57BL/6(N). CCR5KO/CCL17KO mice were obtained by crossing CCR5KO mice to CCL17KO mice. All mice were regularly tested for their KO exploiting PCR.

### Antibodies and flow cytometry

Cells were stained with the following fluorochrome-conjugated antibodies from BD Pharmingen, Biolegend, Novus Biologicals or eBioscience: CD3, CD4, CD8, CD11c, CD25, CD44, CD69, F4/80, IFNγ, IL-2, CD40, CD80, CD86, MHC II. Intracellular staining was performed after adding SIINFEKL peptide at a concentration of 20 μg per ml and Golgi Plug (BD biosciences) at a concentration of 1 μl per ml for 4 hours at 37°C to the culture. Cells were stained for 20 min with CD8 PE, then fixed for 15 min at 4°C with 2% (vol/vol) paraformaldehyde in PBS. After washing, cells were permeabilized in saponin buffer (0.5% of saponin in FACS buffer). Intracellular staining was performed with an APC‐coupled anti‐mouse IFNγ or IL-2 antibody (1:200) in saponin buffer for 30 minutes. All staining steps were supplemented with unconjugated rat immunoglobulin G or human IgG to exclude unspecific binding.

In some experiments, sorting of CFSE proliferation peaks was performed, followed by FACS analysis.

For intracellular staining of Eomes (ThermoFisher), GranzymeB (ThermoFisher), CD127 (BioLegend), cMyc (Novus Biologicals), GATA3 (BioLegend) and KLRG1 (BioLegend), cells were stained intracellularly using BD Pharmingen™ Transcription Factor Buffer Set and/or Foxp3/Transcription Factor Staining Buffer from eBioscience (now ThermoFisher).

For cytokine analysis in cell culture supernatants, commercial enzyme-linked immunosorbent assay kits were used (Invitrogen). OVA-specific CTLs were detected by allophycocyanin-conjugated iTAg MHC class I mouse tetramers (Beckman Coulter). For determination of CCL3 and CCL5 in supernatants, a multiplexed bead-based immunoassay was used following the manufacturer’s instructions (BD Biosciences).

### Isolation of splenic dendritic cells

Spleens were removed and digested by perfusion with 0.4 mg collagenase (from Clostridium histolyticum, Sigma Aldrich) and 100 μl DNAse (from bovine pancreas, Sigma Aldrich) per ml of PBS. After incubation for 20 minutes at 37°C, spleens were homogenized through a metal cell strainer and resuspended in PBS + 0,1% BSA (GE Healthcare). For DC purification, cells were incubated with anti-CD11c-conjugated magnetic microbeads (Miltenyi Biotec) and positively selected with a MACS column according to manufacturer’s instructions.

### Isolation and CFSE labeling of CD8^+^ T cells

Spleen and/or lymph nodes of OT-I, C57BL/6(N) wildtype or knockout mice were extracted and homogenized through a metal cell strainer. After centrifugation, CD8^+^ T cells were isolated by magnetic cell sorting using a CD8^+^ T cell negative selection KIT (Miltenyi Biotec). For carboxyfluorescein diacetate succinimidyl ester (CFSE; Life Sciences)-labeling, 10x10^6^ to 20x10^6^ cells per ml were suspended in PBS, followed by the addition of CFSE to a final concentration of 5 μm, for 10 min at 37°C, as described previously (17). Purity was always over 85% of viable lymphocytes.

### *In vitro* and *in vivo* cross-priming assay

For *in vitro* cross-primimg assays, 1 x 10^5^ CFSE-labeled CD8^+^ T cells were co-cultured with DCs at a ratio of 2:1 in 96‐well plates in a total volume of 200 μl/well at 37°C, with a relative humidity of 90% and a CO_2_ content of 5%. Before co-cultivation, DCs were pulsed either with SIINFEKL peptide (20 μg per ml for 20 minutes at 37°C; ANA SPEC Inc.) or with OVA (1 μg/ml for 2h at 37°C). In some experiments, no DCs but anti-CD3/CD28 beads (Invitrogen) were added to induce antigen-independent CTL activation. After 3 days of co-culture, proliferation and activation of CD8^+^ T cells was cytofluorometrically assessed by CFSE dilution and the division index was calculated using FlowJo10.0 software.

For *in vivo* cross-priming assays, 1 x 10^6^ CFSE-labeled CD8^+^ T cells were transferred into naïve recipients, which were then treated with OVA plus GC and/or CpG. 12-50 hours later, mice were sacrificed, spleens extracted, and single-cell suspensions stained for CD3, CD8, CD25, CD69, or cytokines and cytofluorometrically analyzed.

### *In vivo* cytotoxicity assay

*In vivo* cytotoxicity assays were performed as previously described (17). Briefly, splenic cell suspensions were pulsed for 15 min at 37°C with OVA peptide (SIINFEKL; 2 μg/ml) and labeled with 1 μM CFSE (CFSE^hi^ cells) or were not pulsed with peptide and were labeled with 0.1 μM CFSE (CFSE^lo^ cells). Both target cell types (0.5x10^7^ each) were injected intravenously. 3-7 days earlier, recipient mice were vaccinated with OVA and different adjuvants, as indicated in the results and in the figures. After 4 h, the target cells survival in the spleen was analyzed by flow cytometry. Specific lysis was calculated with the formula: % specific cytotoxicity = 100 − ((CFSE*hi*/CFSE*lo*)_sample_/(CFSE*hi*/CFSE*lo*)_control_) x 100.

### Bioluminescence imaging of AdLGO infected animals

AdLGO was a kind gift from Dirk Wohlleber and Percy Knolle (30). The viral load in C57BL/6(N) mice was quantified by *in vivo* bioluminescence using the real-time IVIS Imaging System 200 (Xenogen Corp., Alameda, CA, USA), as described elsewhere (30–32). Briefly, mice were infected i.v. with 5x 10^5^ PFU AdLGO and analyzed for bioluminescence measurement starting 24 h later. Mice were injected i.p. with 2.5 mg luciferin (S039; Synchem) and anesthetized with isoflurane 5 min before quantification of bioluminescence. Data were analyzed using Living Image 2.50 software (Xenogen Corp.).

### MCMV infection and quantification of infectious virus in host tissues

Bacterial artificial chromosome-derived viruses mCMV-Δm157-SIINFEKL (33) lacking the m157 activatory viral ligand for Ly49H^+^ NK cells (34), here referred to as mCMV-SIINFEKL, and mCMVΔm06/m152-SIINFEKL (35) lacking immune-evasion proteins m06 and m152 were prepared as high titer viral stocks from infected murine embryonic fibroblasts by sucrose-gradient ultracentrifugation as described (36). Subsequently mice were infected i.v. with 1x10^6^ PFU of tissue culture-derived mCMV in 200 µl of PBS. In experiments using mCMVΔm06/m152-SIINFEKL, NK cells were depleted as described 24 h prior to infection.

### Quantification of viral genomes and infectious virus in host tissues

For quantitative PCR, DNA was extracted from 9-12 mg of liver tissue with the viral DNA-OLS kit (Omni Life Science). Serial dilutions of pAd-Track plasmids were used as a standard. For each sample, 4 μL LightCycler FS DNA Master Plus SYBR Green I (Roche), 12 μL double distilled H_2_O, 1 μL forward (5′ TAAGCGACGGATGTGG 3′) and 1 μL reverse (5′ CCACGTAAACGGTCAAAG 3′) primers (20 μM) and 2 μL DNA were mixed and used for measurement with a LightCycler (Roche).

*In vivo* infectivity of mCMV was determined from homogenates of infected organs by standard plaque assay on MEF under conditions of centrifugal enhancement of infectivity (36).

### Statistical analysis

Comparisons were made as indicated with the Mann–Whitney test, Student´s *t* test (unpaired, two-tailed) with Welch’s correction, or two-way ANOVA, Dunnet´s, Bonferroni or Tukey post-test as indicated, using Graph Pad Prism 10 (Graph Pad Software, San Diego, CA).

## Results

### Combining the adjuvants CpG and αGC not only amplifies, but also accelerates the activation of CTL and clearance of viral infections

**Figure 1:**
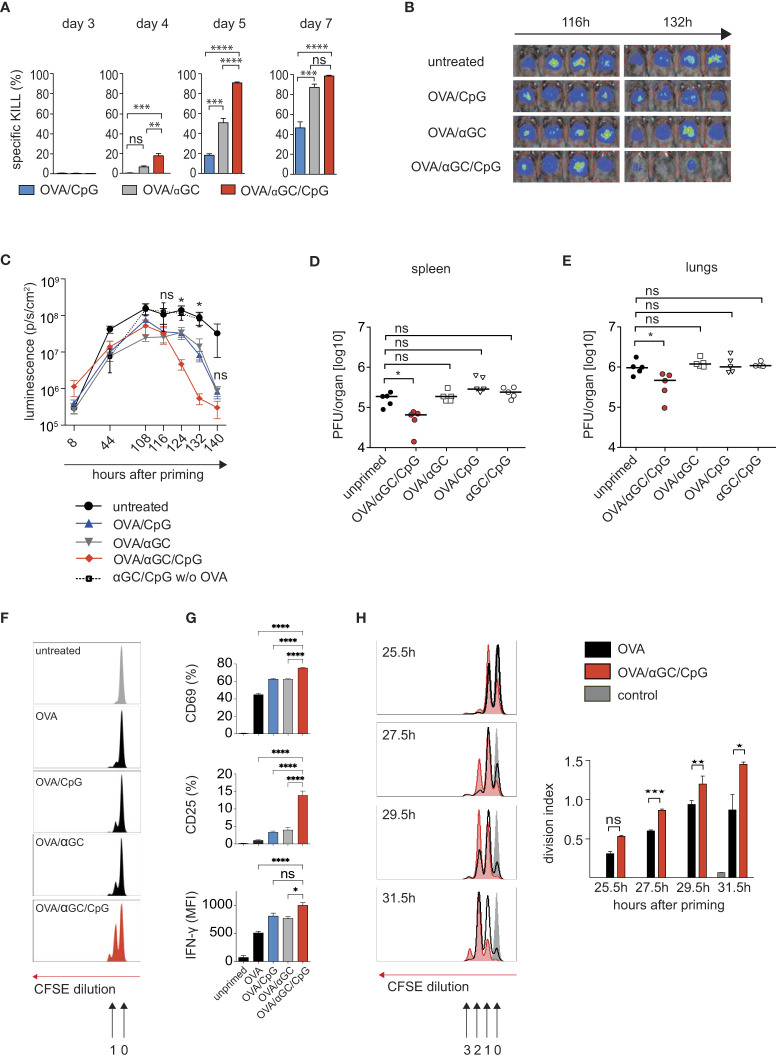
Combining the adjuvants CpG and αGC not only amplifies, but also accelerates the activation of CTL and clearance of viral infections. **(A)** *In vivo* OVA-specific cytotoxic response in the spleen after i.v. vaccination of C57BL/6 mice with OVA plus αGC, CpG or both adjuvants. **(B, C)** Representative luminescence images **(B)** on days 4.5 and 5.5 after i.v. vaccination and **(C)** bioluminescence as time course over 140 h in mice infected with AdLGO that were vaccinated i.v. with OVA plus the adjuvants CpG, αGC, or both 12 h after infection. **(D, E)** Viral organ titers at day 4 post infection in spleen and lungs of wt mice i.v. infected with 1x10^6^ PFU mCMV-SIINFEKL and vaccinated i.v. with OVA together with the indicated adjuvant combinations. The median is indicated by horizontal bar. **(F, G)** Proliferation profiles **(F)**, activation markers and IFNγ production **(G)** by CFSE-labeled OT-I cells transferred into wt mice and analyzed 24 h after i.v. vaccination with different adjuvant combinations. **(H)** Histograms and division indices of CFSE-labeled OT-I cells transferred into wt recipients that on the following day were vaccinated i.v. with OVA or OVA/αGC/CpG. Cell cycle divisions were measured 25.5, 27.5, 29.5, and 31.5 h after vaccination. Numbers indicate the division cycle (approx. 4-4.5h). Unprimed controls are shown in grey. Bars show mean and s.e.m. of one representative experiment with 3-4 mice/group of three repetitions. *P* values throughout were calculated using unpaired Student´s *t* test (two-tailed, **H**) or two-way ANOVA/Bonferroni multi-comparison test **(A, D, E, G)** or Mann Whitney test **(C)**. *p </= 0.05, **p </= 0.01, ***p </=0.001, ****p </= 0.0001. ns, not significant.

To clarify whether adjuvants that induce signal 0 chemokines can accelerate CTL activation, we used a well-characterized vaccination model based on the model antigen OVA, which together with the TLR9 ligand CpG induces Th cell-dependent classic cross-priming (5, 6). The combination with the NKT cell ligand αGC causes NKT-cell mediated alternative cross-priming (17). Both adjuvants induce signal 0-chemokines that attract naive CTL to the DCs. Using the optimal CpG and αGC doses (17), we confirmed that vaccination with OVA and both adjuvants induced higher cytotoxicity than only one adjuvant (**Figure 1A**). Importantly, we here noted that higher cytotoxicity was detectable at earlier time points, such as on days 4 and 5 (**Figure 1A**), suggesting that the adjuvant combination not only amplified the CTL response, but also accelerated its inception. Rapid CTL activation theoretically should be useful for therapeutic vaccination strategies *after* viral infection. To mimic such a “post-exposure vaccination” situation, we employed two CTL-dependent murine infection models: First, we used a recombinant adenovirus expressing OVA and luciferase (AdLGO), which causes hepatitis that can be monitored by luminescence imaging (30, 32). 12 hours after infecting mice with AdLGO, we vaccinated them with OVA plus adjuvant combinations. Indeed, mice vaccinated with OVA/αGC/CpG showed lower virus loads after 124 and 132 hours than non-vaccinated mice or mice that had received only one adjuvant (**Figures 1B, C**), indicating faster clearance of the infection. Mice injected with both adjuvants but without OVA failed to clear the viral infection after 140 hours (**Figures 1B, C**), indicating antigen-specificity of protection and excluding that only innate immune mechanisms were involved.

We decided to confirm our findings in a second virus infection, using murine cytomegalovirus (mCMV), a well-characterized model for human cytomegalovirus infection, whose control is also dependent on the induction of an robust CD8 T cell response (37). The importance of the CD8 T cell response has been shown in clinical and experimental settings (38), especially in recipients of hematopoietic cell transplantation (39). The lungs represent a highly relevant organ site of CMV pathogenesis in the immunosuppressed host, where the most critical manifestation of CMV diseases, interstitial pneumonia, occurs (40–42).

We infected mice with mCMV-SIINFEKL, in which an immunodominant viral CTL epitope was replaced with the OVA-peptide SIINFEKL (35). Then we immunized mice with OVA/αGC/CpG or with only one adjuvant plus OVA, and determined viral titers in spleen and lungs 4 days later. Indeed, only mice immunized with OVA/αGC/CpG showed a reduced viral load on day 4 post infection, but not the mice vaccinated with one adjuvant alone (**Figures 1D, E**). Taken together, these experiments indicated that the combination of the adjuvants αGC and CpG had allowed earlier CTL activation and faster post-exposition prophylaxis in two model virus infections.

### Antigen-specific CTL commence proliferation earlier in response to the adjuvant combination CpG/αGC

To track antigen-specific CTL after vaccination, we used OT-I cells that express a transgenic OVA-specific cell receptor that recognizes SIINFEKL in the context of the MHC I molecule K^b^. We labeled OT-I cells with the fluorescent dye CFSE, whose dilution indicates cell proliferation (43). Using that system, we had previously shown that OT-I cells commence proliferation in response to OVA without adjuvant after approximately 20 hours and then complete a cell cycle each 4.5 hours (43). Here, we used that system to compare the number of cell cycles CTL can accomplish until 24 hours after vaccination. OT-I cells responding to OVA/αGC/CpG immunization, had accomplished more than one division cycle, in contrast to vaccination with one or no adjuvant (**Figure 1F**), confirming faster CTL activation. Furthermore, after 24 hours, more OT-I cells responding to both adjuvants produced the effector cytokine IFNγ and expressed the IL-2 receptor CD25 and the early activation marker CD69 (**Figure 1G**), which is upregulated after encountering DCs that express cognate antigen. This supported earlier encounters with antigen-presenting DCs as we previously showed (17).

When we compared the CFSE proliferation profiles of OT-I cells at additional early time points after vaccination, we noted that more OT-I cells activated in the presence of both adjuvants had completed the first cell cycle after 25.5 hours than those responding to OVA alone (**Figure 1H**). After 27.5 and 29.5 hours, OT-I cells responding to OVA/αGC/CpG showed a significant lead and had completed more cell cycles. After 31.5 hours, some of them had completed the third cell cycle, but none of the OT-I cells responding to OVA alone had done so (**Figure 1H**). Although the time span that can be examined with the CFSE method is too short for more precise calculations, the progression of OT-I cells through the proliferation peaks was consistent with a head start of 4-4.5 hours for OT-I cells activated by OVA/αGC/CpG, followed by cell cycles at a constant speed of 4.5 hours, regardless of whether adjuvants were used or not (43). In other words, CTL were activated earlier when both adjuvants were applied, but did not perform accelerated cell cycles.

### Signal 0-chemokines mediate faster CTL activation in response to CpG/αGC

**Figure 2:**
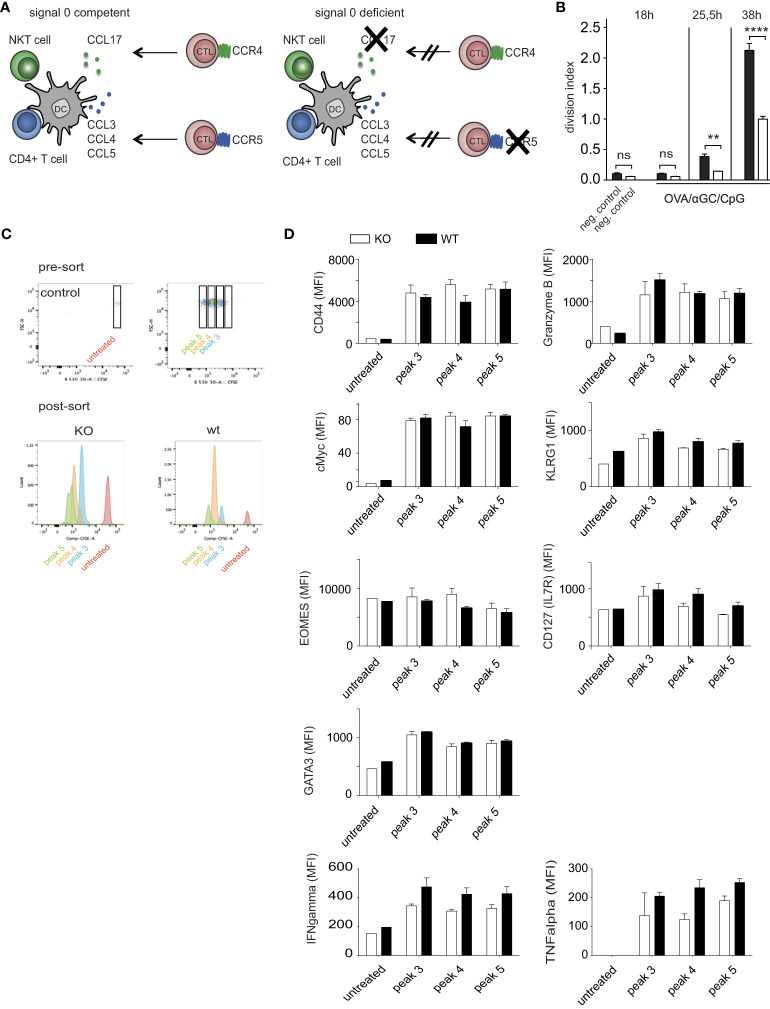
Signal 0-chemokines mediate faster CTL activation in response to CpG/αGC. **(A)** Experimental scheme of chemokine-competent (left) and chemokine-deficient (right) mice. **(B)** Division indices of CFSE-labeled wt OT-I or OT-I.CCR5^-/-^ cells transferred into wt mice or CCL17-deficient mice analyzed at 0, 25.5, and 38 h after i.v. vaccination with OVA/αGC/CpG. **(C)** Proliferation of CFSE-labeled wt OT-I or OT-I.CCR5^-/-^ cells transferred into wt mice or CCL17-deficient mice analyzed at 50 h after i.v. vaccination with OVA/αGC/CpG. Sorting of different CFSE proliferation peaks was performed, followed by intra/extracellular staining for FACS analysis. Corresponding division cycles in the presence (wt) and absence (ko) of signal 0-chemokines shown in the same color. **(D)** Flow cytometric analysis of various functional parameters of OT-I cells in corresponding proliferation peaks 3-5, as indicated in **(C)**. Results are shown from one representative experiment of three repetitions with 3-4 mice/group. Bars show mean and s.e.m. of this representative experiment with 3-4 mice/group. White bars = KO, black filled bars = wildtype. *P* values throughout were calculated using two-way ANOVA/Bonferroni multi-comparison test **(D)** or Newman-Keuls **(B)**. **p </= 0.01, ****p </= 0.0001. ns, not significant. None of the analyzed parameters in **(D)** achieved significance.

Chemokines can guide CTL faster toward antigen-presenting DCs (15–17). We therefore investigated whether the earlier OT-I cell activation in response to OVA/αGC/CpG (**Figure 1**) was mediated by signal 0-chemokines. This required using OT-I cells that cannot sense such chemokines. However, such cells cannot be generated by crossing CCR4^–/–^ to CCR5^–/–^ mice, because these chemokine receptors are located adjacently on the same chromosome. Instead, we transferred OT-I.CCR5^–/–^ cells into mice deficient for CCL17 (experimental scheme in **Figure 2A**). As CCL17-deficient mice, homozygous CCL17- eGFP knock-in mice were applied in which both Ccl17 genes were replaced with a gene encoding enhanced green fluorescent protein (eGFP), leaving the Ccl17 promoter in place (17, 29). Cross-priming DCs produce only CCL17, but not CCL22 (17), the other known CCR4 ligand. Indeed, at 25.5 and at 38 hours after immunization with OVA/αGC/CpG, fewer OT-I.CCR5^–/–^ cells had divided in CCL17^–/–^ mice compared to wildtype (wt) OT-I cells transferred into wt mice (**Figure 2B**). We ruled out defects in effector functions in OT-I.CCR5^–/–^ cells (**Supplementary Figures 1A–E**) and of DCs in CCL17-deficient mice (17). These findings confirmed that signal 0-chemokines were responsible for the CTL head start after vaccination with OVA/αGC/CpG.

The effect of signal 0-chemokines on OT-I cell proliferation (**Figure 2B**) suggested that also CTL functionality might be affected. This question could not be answered by comparing signal 0-deficient and competent OT-I cells at the same time after priming, because the head start of the latter cells may allow them to differentiate further and to develop effector functions. To avoid this problem, we compared functional parameters between signal 0-deficient and competent OT-I cells in corresponding proliferation peaks (**Figures 2C, D**; **Supplementary Figure 2**), as these presumably represented cells activated the same period of time ago. Indeed, many parameters showed comparable values for the corresponding peaks (**Figure 2D**; **Supplementary Figure 2**). In particular, the expression of the CTL activation marker CD44, of the transcription factors cMyc [parameter for the proliferative potential of CTL (44)], the transcription factors GATA3 and Eomes (regulating CTL differentiation in response to cytokines), of the central memory marker IL7R (CD127), and of the cytotoxic function markers KLRG1 and Granzyme B (GrzB) were similar between signal 0-competent and -deficient OT-I cells of corresponding cell cycle peaks. We also analyzed the expression of the key effector cytokines TNFα and IFNy for each peak and found no significant differences (**Figure 2D**). These results argue against major differences in the functionality of CTLs by signal 0 chemokines, at least at these early time points after activation.

### Signal 0-chemokines synergistically accelerate activation of CTL in a normal T cell repertoire by 1-2 days

**Figure 3:**
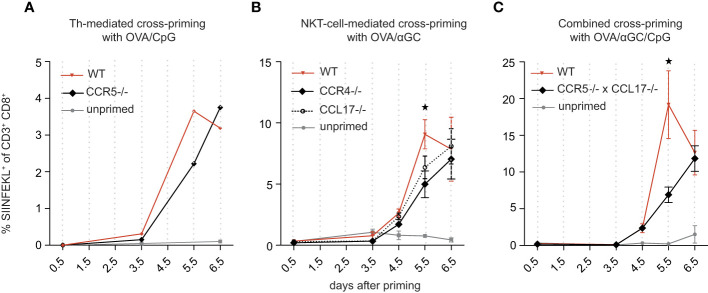
Signal 0-chemokines synergistically accelerate activation of CTL in a normal T cell repertoire by 1-2 days. **(A–C)** Percentage of antigen-specific (SIINFEKL+) CTL in wt and CCR5^-/-^mice vaccinated i.v. with OVA/CpG **(A)**, in wt, CCR4^-/-^, and CCL17^-/-^ mice vaccinated i.v. with OVA/αGC **(B)**, and in wt and CCR5/CCL17-deficient mice vaccinated i.v. with OVA/αGC/CpG **(C)**, quantified over several days in an endogenous T cell repertoire. A shows pooled results of 4-5 mice due to little amounts of antigen-specific CTL. Results in **(B, C)** are shown from one representative experiment of three repetitions with 3-4 mice/group. Bars show mean and s.e.m. “Unprimed” means that these mice have not been vaccinated with neither OVA nor adjuvant. *P* values were calculated using two-way ANOVA/Bonferroni multi-comparison test [**(A–C)**, on day 5.5, wt versus KO group]. *p </= 0.05.

The head start of OT-I cells of 4-4.5 hours (**Figure 1**) may appear small, but this period was determined in experiments where many OT-I cells had been adoptively transferred. The probability that some of these numerous OT-I cells encounter relevant DCs is higher than in a normal T cell repertoire, where CTL frequencies for a given antigen are orders of magnitude smaller. We speculated that signal 0 may be more effective for such low CTL frequencies and quantified the signal 0-mediated CTL head start in a naïve T cell-repertoire. To this end, we vaccinated CCR5-deficient mice with OVA plus CpG (**Figure 3A**) and CCR4-deficient mice with OVA plus αGC (**Figure 3B**), and found in both cases a delay in the generation of antigen-specific CTL of 0.5-1 days compared with signal 0-competent control mice, supporting the hypothesis that signal 0 is more effective in a normal T cell repertoire.

To test for synergy, we generated mice deficient for both CCR5 and CCL17. We first excluded quantitative and qualitative immune defects of these mice and detected no activation or proliferation deficit in CCR5 knockout OT-Is (**Supplementary Figure 1**). As previously described (17), CCL17 knockout OT-Is are equally potent in proliferation as wildtype OT-Is, indicating that CCL17 has no major effects on T cell proliferation. Notably, in double-knockout DC, no deficit in DC activation could be detected. (**Supplementary Figures 3A–H**). When we vaccinated CCR5/CCL17-deficient mice with OVA/CpG/αGC, OVA-specific CTL activation was delayed by approximately 1-2 days compared with signal 0-competent controls (**Figure 3C**), consistent with the combined delays of 0.5-1 days in mice lacking only one signal 0 chemokine system (**Figures 3A, B**). Thus, the two signal 0-chemokine systems synergistically accelerated CTL activation in a normal T cell repertoire, resulting in a head start of up to 2 days.

### Signal 0-chemokines accelerate viral clearance after post-exposure vaccination

**Figure 4:**
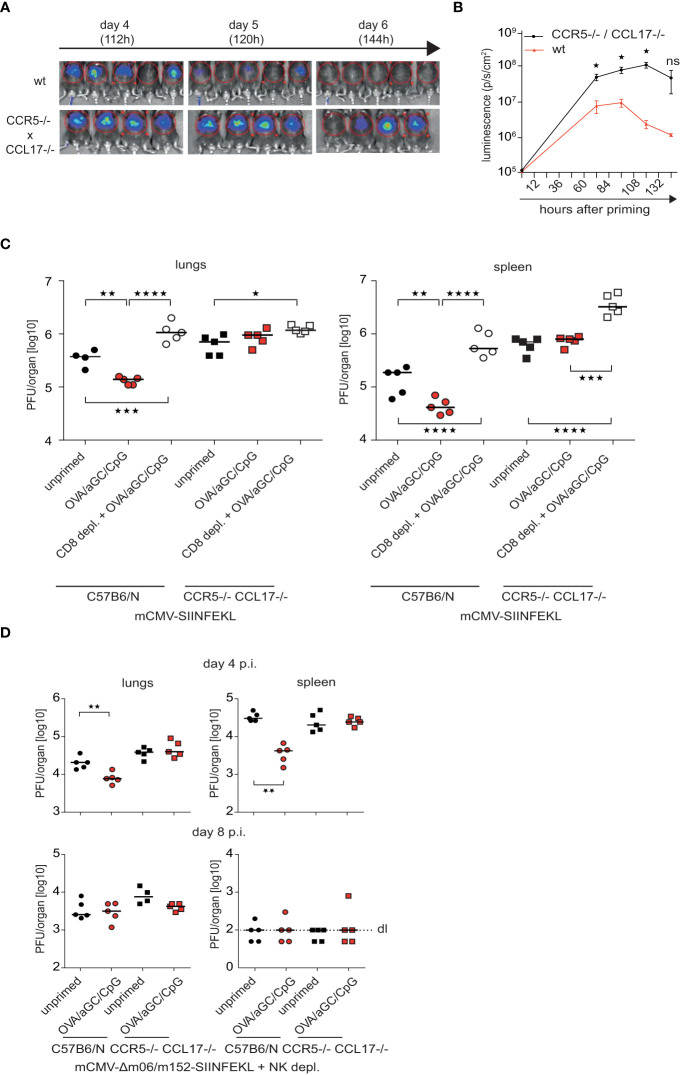
Signal 0-chemokines accelerate viral clearance after post-exposure vaccination. **(A, B)** Luminescence images **(A)** and luminescence **(B)** of CCR5/CCL17-deficient mice and wt mice i.v. vaccinated with OVA/CpG/αGC in the experimental set-up described in **Figures 1B, D** Graphs show mean and s.e.m. from independent, representative experiments with 3-5 mice/group of three repeats. *P* values were calculated using Mann-Whitney test (two-tailed, WT vs. KO group). *p </= 0.05, **p </= 0.01, ***p </=0.001, ****p </= 0.0001. **(C)** Viral titers in lungs and spleen in wt (circle) or CCR5/CCL17-deficient mice (square) mice, which had been infected i.v. with 1x10^6^ PFU of mCMV-SIINFEKL, depleted of CD8^+^ cells (open symbols), vaccinated 6h post infection with OVA/αGC/CpG (red and white symbols) or left unvaccinated (“unprimed”, black symbols). Viral titers were determined by plaque assay on day 4 p.i. **(D)** Viral titers in lungs and spleen in wt (circle) or CCR5/CCL17-deficient mice (square) mice, which had been infected i.v. with 1x10^6^ PFU of mCMV-Δm06/152-SIINFEKL and depleted of NK cells. Viral titers were determined by plaque assay on day 4 and 8 p.i. Each symbol represents an individual mouse; horizontal bars indicate median values. *P* values were calculated using two-way ANOVA/Bonferroni multi-comparison test. *p </= 0.05, **p </= 0.01, ***p </=0.001, ****p </= 0.0001. ns, not significant.

The head start of 1-2 days can theoretically explain why the αGC/CpG adjuvant combination was able to induce faster control of adenovirus and mCMV infections after post-exposure vaccination (**Figure 1**). To investigate whether this was mediated by signal 0-chemokines, we infected CCR5/CCL17-deficient or competent mice with AdLGO, then vaccinated them with OVA/CpG/αGC and determined the viral load by luminescence imaging. Indeed, signal 0-deficient mice cleared the adenovirus with a delay of almost 1.5–2-days (**Figures 4A, B**), consistent with the CTL kinetics in a normal T cell repertoire we had determined above (**Figure 3**).

Next, we tested whether signal 0 chemokines can also improve post-exposure vaccination against mCMV. To this end, we first infected immunocompetent mice with mCMV-SIINFEKL and after 6 hours immunized them with OVA/αGC/CpG. After 4 days, we determined viral titers in spleen and lungs. Immunized mice showed a reduced viral load unless CTL were depleted (**Figure 4C**), indicating success of post-exposure vaccination and confirming the critical role of CTL in virus control. Viral titers were much higher in CCR5/CCL17-deficient compared to competent mice (**Figure 4C**), confirming that signal 0 chemokines can accelerate the effectiveness of therapeutic vaccination against mCMV. Viral titers did not differ in immunized, non-immunized and CTL-depleted CCR5/CCL17-deficient mice at this early time-point (**Figure 4C**), verifying that the success of post-exposure vaccination was dependent on CTL and signal 0-chemokines.

The mCMV expresses the proteins m06 and m152, which interfere with the cell surface presentation of peptide-loaded MHC I complexes, allowing evasion of recognition by antiviral CTL (8, 9, 35, 45). We speculated that signal 0 might be even more effective in viruses lacking such immune escape strategies. We tested this idea using a mCMV mutant that lacks m06 and m152, and thus induces stronger CTL responses than mCMV-SIINFEKL. Consequently, titers of this virus mutant were an order of magnitude lower than those of mCMV-SIINFEKL (**Figures 4C, D**). Importantly, post-exposure vaccination established antiviral protection against the mutant virus only if signal 0 was operational (**Figure 4D**). This finding also excluded that signal 0 might operate by preventing m06 and m152-mediated immune evasion of mCMV infection. On day 8 after infection, differences in the viral titers between signal 0-competent and –deficient mice were no longer evident (**Figure 4D**), indicating that the antiviral defense by CTL can principally be established without signal 0, but at the cost of a delay of days. Taken together, these findings indicated that signal 0-chemokines accelerated CTL responses in two viral infection models, suggesting applicability in the improvement of post-exposure vaccination strategies.

## Discussion

We here demonstrate that adjuvants that induce synergistic signal 0-chemokines accelerate the establishment of antiviral CTL immunity. The speed by which such protection is established represents a distinct vaccine quality that has so far received less attention than its strength or duration, which are critical for prophylactic vaccinations. We found that mice deficient for the two known signal 0-chemokine systems mounted a CTL response of a magnitude comparable to that in wildtype mice, but with a delay of 1–2 days. We previously showed that signal 0-chemokines allow naïve CTL to find antigen-bearing DCs faster (17), explaining mechanistically why signal 0-competent CTL were activated earlier and completed more cell cycles in the same observation time after vaccination.

These findings imply that caution is warranted when T cell responses *in vivo* are compared at one single time point after vaccination. If signal 0-chemokines differ between experimental groups, then the head start of CTL in the signal 0-competent group allows these CTL to expand and differentiate more than those in the signal 0-deprived group. This might be misinterpreted as functional inferiority of the latter CTL, but in fact, they only lagged behind. Consistent with this interpretation, functional parameters including cell surface activation markers, transcription factors or effector cytokines did not differ when signal 0-competent and -defective CTL were compared that had completed the same number of cell cycles.

The rationale behind signal 0-chemokines may be speeding up the establishment of protection against primary viral infections. This “jump-start” accelerated by 1–2 days the defense of two virus infection models, namely adenoviral hepatitis and mCMV, which possess clinically relevant human disease correlates (37, 46, 47). Other adjuvants may exist that induce additional signal 0 chemokine systems that target receptors other than CCR4 and CCR5, which may be used to further improve the vaccination strategy reported here.

Gaining two days is not critical for prophylactic vaccinations. However, for post-exposure vaccination after viral exposure, even a single day can make an important difference because of the rapidity of viral replication. This is especially true for immunosuppressed patients, for example in acquired immune deficiency syndrome (AIDS) (46) or after hematopoietic stem cell and solid organ transplantation (37, 48). Herpes viruses, especially CMV, pose a particularly important threat for the immunocompromised patients (49). Thus, an earlier protection against such viruses may reduce morbidity by limiting the exponential viral growth and spread within the host (50) in the vulnerable phase until protective antibodies are available (51). Signal 0-optimized vaccination may also be helpful in individuals who have been exposed to infected persons during seasonal viral epidemics, or shortly before or during trips into areas affected by endemic infections, i.e. in the tropics where hemorrhagic fevers cause recurrent epidemies. A recently published analysis of a vaccination trial in the 2018 Ebola outbreak in central Africa, where confirmed contacts of cases and frontline healthcare workers were immunized within 2 weeks, concluded that “early implementation of vaccination was critical”, as a delay of only a few days compromised vaccine effectivity substantially (1). Finally, an accelerated induction of humoral and cellular immunity could be of high importance during pandemics, where abbreviating the infectiousness of patients by only a few days could profoundly impact on the worldwide highly dynamic progression of the virus. Theoretically, our approach might also be applicable during COVID-19 pandemics. Thus, post-exposure prophylactic strategies may benefit from adjuvants optimized for inducing synergistic signal-0-chemokines that speed up CTL induction.

## Data availability statement

The original contributions presented in the study are included in the article/**Supplementary Material**. Further inquiries can be directed to the corresponding author.

## Ethics statement

*In vivo* experiments were approved by the Local Animal Care Commission of North Rhine Westphalia (permit number 84-02.04.2016.A102 and 84-02.04.2014.A140) or by the ethics committee of the Landesuntersuchungsamt Rheinland-Pfalz (permit number 23177-07/G11-1-004). The study was conducted in accordance with the local legislation and institutional requirements.

## Author contributions

AH: Conceptualization, Data curation, Formal analysis, Funding acquisition, Investigation, Methodology, Project administration, Resources, Validation, Visualization, Writing – original draft, Writing – review & editing. NL: Conceptualization, Funding acquisition, Data curation, Formal analysis, Investigation, Methodology, Project administration, Resources, Validation, Visualization, Writing – original draft, Writing – review & editing. CF: Data curation, Formal analysis, Investigation, Methodology, Validation, Writing – review & editing. JB-G: Data curation, Formal analysis, Investigation, Methodology, Validation, Writing – review & editing. NG: Writing – review & editing, Validation. PB: Writing – review & editing, Funding acquisition, Supervision. CK: Funding acquisition, Supervision, Writing – review & editing, Conceptualization, Formal analysis, Investigation, Project administration, Resources, Validation, Writing – original draft.
