# Supplementary material for: Rapid protection against viral infections by chemokine-accelerated post-exposure vaccination
Source: Front Immunol. 2024 Jan 29;15:1338499. doi: 10.3389/fimmu.2024.1338499 (PMC10860197; doi:10.3389/fimmu.2024.1338499)
Supplement: Supplementary file 1 [file DataSheet_1.pdf]

Fig. S1

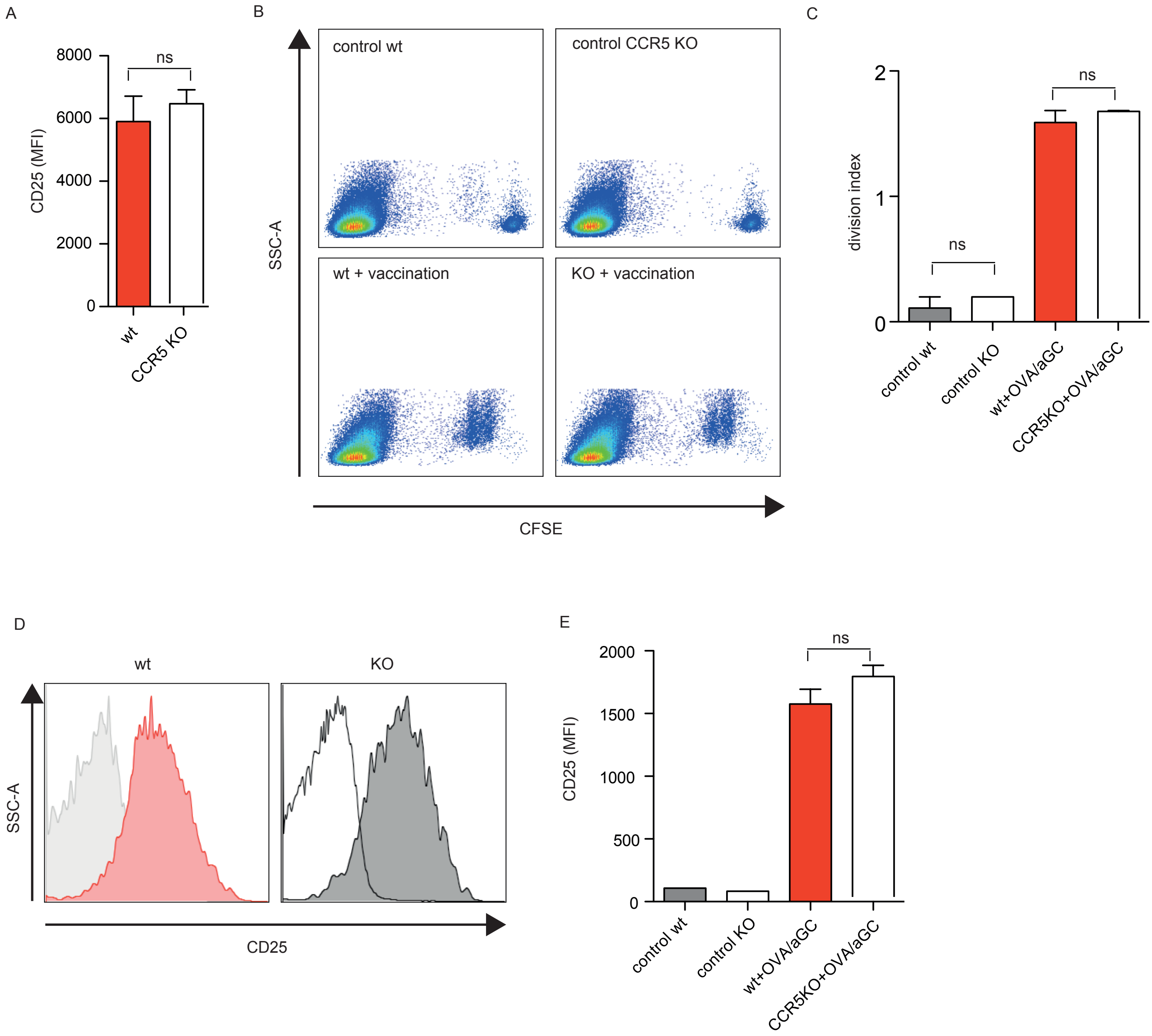

A
CD25 (MFI)
8000
6000
4000
2000
0
ns
wt
CCR5 KO
B
control wt
control CCR5 KO
wt + vaccination
KO + vaccination
SSC-A
CFSE
C
division index
2
1
0
ns
ns
control wt
control KO
wt+OVA/aGC
CCR5KO+OVA/aGC
D
wt
KO
SSC-A
CD25
E
CD25 (MFI)
2000
1500
1000
500
0
ns
control wt
control KO
wt+OVA/aGC
CCR5KO+OVA/aGC

Fig. S2

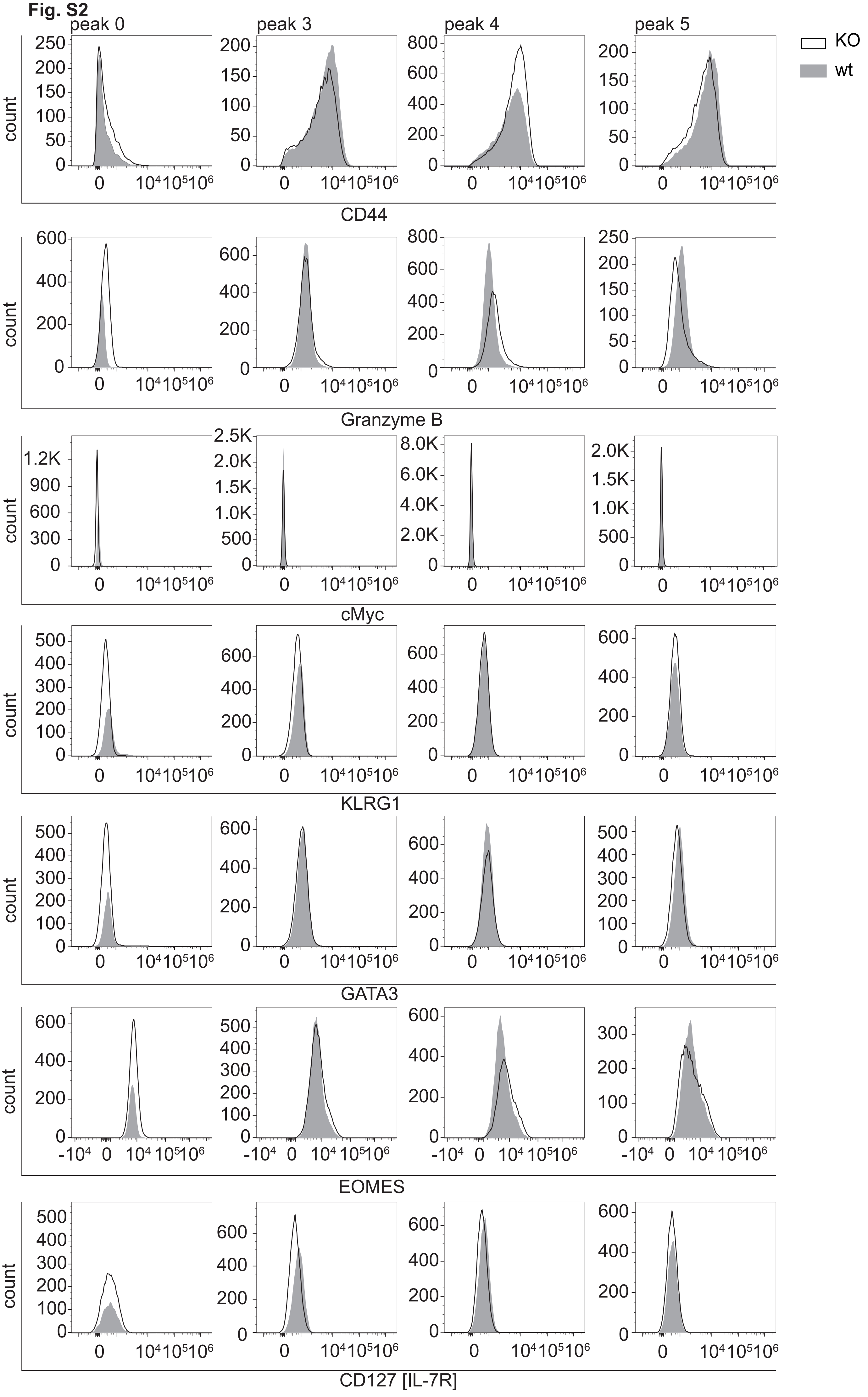
peak 0
peak 3
peak 4
peak 5
KO
wt
count
CD44
Granzyme B
cMyc
KLRG1
GATA3
EOMES
CD127 [IL-7R]

Fig. S3

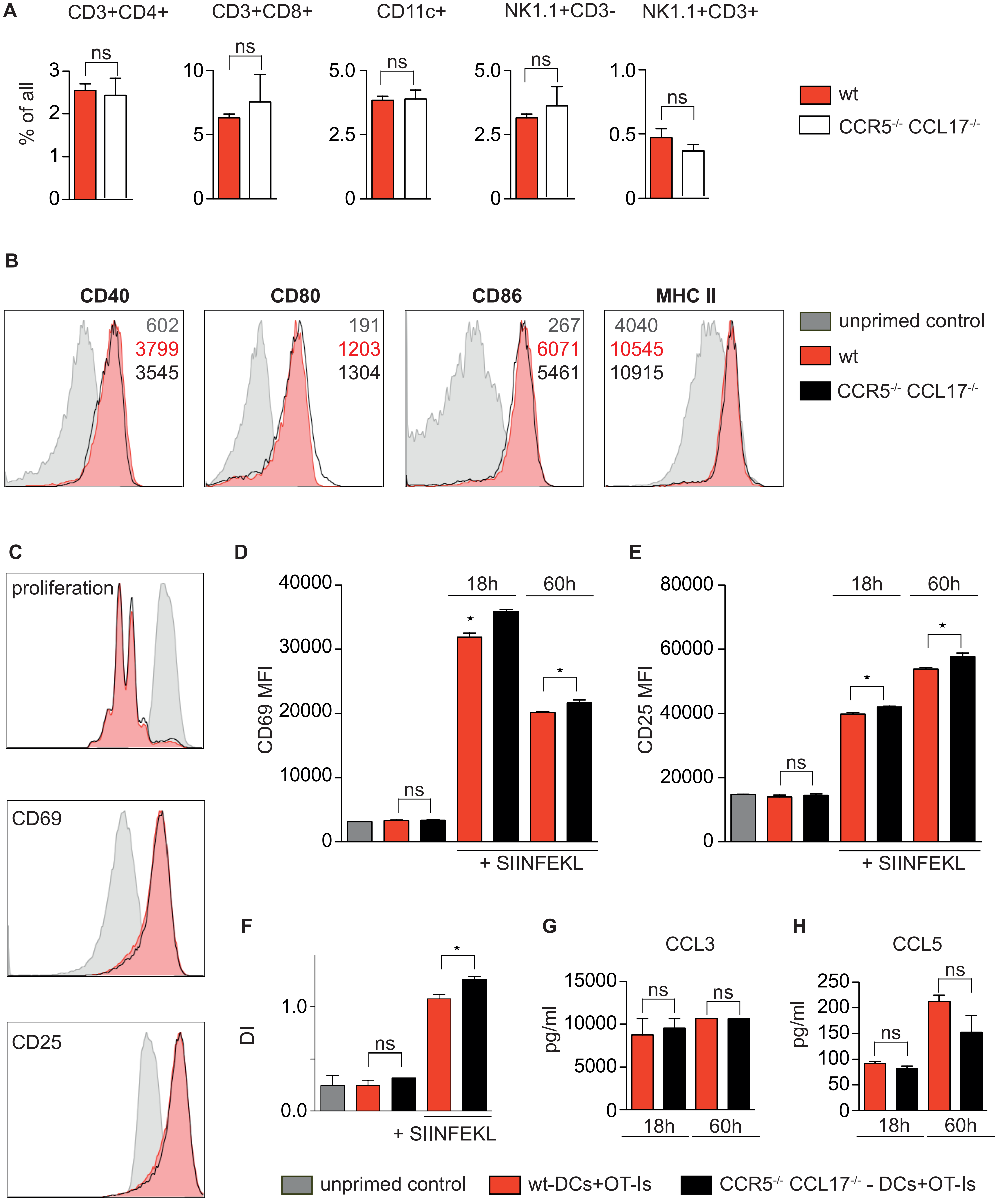
A
CD3+CD4+
CD3+CD8+
CD11c+
NK1.1+CD3-
NK1.1+CD3+
% of all
ns
wt
CCR5-/- CCL17-/-
B
CD40
CD80
CD86
MHC II
602
3799
3545
191
1203
1304
267
6071
5461
4040
10545
10915
unprimed control
wt
CCR5-/- CCL17-/-
C
proliferation
CD69
CD25
D
CD69 MFI
18h
60h
+ SIINFEKL
E
CD25 MFI
F
DI
G
CCL3
pg/ml
H
CCL5
unprimed control
wt-DCs+OT-Is
CCR5-/- CCL17-/- - DCs+OT-Is

Supplementary figures

Figure S1:

OT-I.CCR5$^{-/-}$ cells show no functional defects compared with wt OT-I cells. (A) CD25 expression of CFSE-labeled wt OT.I cells and CCR5$^{-/-}$ OT.I cells 2.5 days after *in vitro* stimulation with anti-CD3/CD28 beads. **(B, C)** Cell divisions and **(D, E)** CD25 expression (both as FACS blots and bar graphs) of CFSE-labeled wt OT.I cells or CCR5$^{-/-}$ OT.I cells 38h after transfer into wt recipient mice vaccinated with OVA/αGC. Bars show mean and s.e.m. of a representative experiment of three repeats. *P* values throughout calculated using unpaired student´s t test (two-tailed). In Fig. 1B, CFSE staining is depicted on the x-axis, SSC-A on the y-axis. In Fig. 1D, CD25 staining is depicted on the x-axis, SSC-A on the y-axis. * *p* </= 0.05, ***p* </= 0.01, ****p* </= 0.001, *****p* </= 0.0001.

Figure S2:

Signal 0-chemokines do not functionally alter CTL early after cross-priming.

Sorting strategy is depicted in Figure 2C. CFSE-labeled wt OT-I cells transferred into wt (wt) or OT-I.CCR5$^{-/-}$ cells transferred into CCL17-deficient mice (KO), followed by vaccination with OVA/αGC/CpG the next day and cytofluorometric analysis after 50h. Depicted are histograms of one representative experiment.

Figure S3:

CCR5$^{-/-}$ / CCL17$^{-/-}$ mice do not show immune cell-intrinsic functional defects

(A) Immune cell subsets of wt and CCR5$^{-/-}$/CCL17$^{-/-}$ mice 20 h after vaccination with OVA/αGC/CpG. **(B)** *In vivo* activation markers of CD11c+CD8+ DCs 20 h after vaccination with OVA/αGC/CpG. Numbers indicate MFI of unvaccinated control group, vaccinated wt mice, and vaccinated KO mice of representative mice. **(C-E)** Proliferation, expression of CD69 and CD25 at 18 and 60 h after coculture of CFSE-labeled wt OT.I cells with either wt DCs or CCR5$^{-/-}$ CCL17$^{-/-}$ DCs *in vitro* with or without the antigenic peptide SIINFEKL. **(F)** Division indices of CFSE-proliferation after 60 h. **(G-H)** CCL3 and CCL5 were quantified from supernatants using LUMINEX technology after 18 and 60 h of coculture. Bars show geometric mean and s.e.m. *P* values throughout were calculated using Student´s *t* test. * *p* </= 0.05, ***p* </= 0.01, ****p* </= 0.001, *****p* </= 0.0001.
